# Evidencing the presence of merkel cell polyomavirus in papillary thyroid cancer

**DOI:** 10.1038/s41598-021-01055-2

**Published:** 2021-11-02

**Authors:** Monir Ghanghareh, Jalal Mosayebi Amroabadi, Seyed Mohammad Tavangar, Shiva Irani, Fatemeh Sakhaee, Morteza Ghazanfari Jajin, Farzam Vaziri, Seyed Davar Siadat, Abolfazl Fateh

**Affiliations:** 1 Department of Biology, Science and Research Branch, Islamic Azad University, Tehran, Iran; 2 Artificial Intelligence and Multi-omics Center (AIMOC), Stavanger, Norway; 3 Chronic Diseases Research Center, Endocrinology and Metabolism Population Sciences Institute, Tehran University of Medical Sciences, Tehran, Iran; 4 Department of Pathology, Dr. Shariati Hospital, Tehran University of Medical Sciences, Tehran, Iran; 5 Departments of Mycobacteriology & Pulmonary Research, Pasteur Institute of Iran, Tehran, Iran; 6 Department of Clinical Biochemistry, School of Medicine, Shahid Sadoughi University of Medical Sciences, Yazd, Iran; 7 Microbiology Research Center (MRC), Pasteur Institute of Iran, Tehran, Iran

**Keywords:** Viral pathogenesis, Tumour virus infections

## Abstract

Merkel cell polyomavirus (MCPyV) infects most people asymptomatically, but recent reports indicate that the virus may be related to carcinogenesis. This study aimed to evaluate the impact of MCPyV on the development of papillary thyroid cancer (PTC). Totally, 1057 samples, including 412 fresh biopsy samples (FBS) and 645 paraffin-embedded PTC biopsy samples (PEBS), and 1057 adjacent non-cancerous samples were assessed for the presence of MCPyV DNA and RNA. MCPyV DNA was positive in 215 (20.3%) of samples, including 126 (30.6%) in FBS and 89 (13.8%) in PEBS. In MCPyV-positive samples, the mean MCPyV copy number was higher in the patients with FBS (2.3 × 10^–1^ ± 0.5 × 10^–1^ copies/cell) compared to PEBS (0.7 × 10^–4^ ± 0.1 × 10^–4^ copies/cell) and adjacent non-PTC normal samples (0.3 × 10^–5^ ± 0.02 × 10^–5^ copies/cell), indicating a statistically significant difference (*P* < 0.001). The *LT*-Ag RNA expression was higher in FBS compared to PEBS, while *VP1* gene transcript was not detected in any samples. Although our findings showed the presence of MCPyV in a subset of PTC Iranian patients, further research is required to confirm these findings.

## Introduction

Papillary thyroid cancer (PTC) is the most frequent form of well-differentiated thyroid cancer, which is the most common type of cancer caused by radiation exposure. PTC, despite its distinctive features, may be overtly or minimally invasive. It seems that these tumors may easily spread to other organs. The well-known risk factors for the PCT development are ionizing radiation exposure, autoimmune thyroid diseases, genetic predisposition, and high iodine intake^1^. The role of some viral infections such as human papillomavirus, polyomaviruses, herpesviruses, and parvovirus B19 in the pathogenesis of PTC has been suggested^2,3^.

For the first time, Merkel cell polyomavirus (MCPyV), that is widespread in the general population, was discovered in Merkel cell carcinoma (MCC)^4^. Since the discovery of virus, MCPyV has been known as a ubiquitous virus that begins asymptomatically in most people from childhood^5^.

Although MCPyV has a definite association with MCC, and 80% of these cancers are MCPyV positive, its possible correlation with several other cancers has recently been a common topic of exploration. There is evidence that in addition to MCC, MCPyV may be related to other types of skin cancer, cervical cancer, lung cancer, extrapulmonary small cell carcinoma, and even certain types of leukemia^4^.

To date, no study has indicated that MCPyV can be associated with PTC. Therefore, the aim of this study was to investigate whether this virus could be one of the possible risk factors for PTC.

## Materials and methods

### Patients and samples collection

In this cross-sectional study, a total of 1057 biopsy samples, including 412 fresh biopsy samples (FBS) and 645 paraffin-embedded PTC biopsy samples (PEBS), and 1057 adjacent non-PTC normal samples were selected from three centers in Tehran, Iran from October 2014 to March 2020. The current study was approved by the Ethical Committee of Pasteur Institute of Iran and was performed according to the 1975 Declaration of Helsinki and relevant local regulations. Also, a written informed consent was obtained from all patients.

### DNA extraction

Deparaffinization was treated with 1 ml xylene to the tissue-section-containing microtubes that were powerfully mixed by vortexing for 15 s. After that, the tubes were centrifuged at 16,000×*g* for 2 min, and the supernatant was discarded (to be repeated three times). Then, the pellet was washed three times by adding 1 ml ethanol 96%, followed by centrifugation at 20,000×*g* for 3 min, after which the supernatant was discarded. The tissue pellet was dried at 37 °C (open tubes) for 40 min until the remaining ethanol has evaporated.

After tissue deparaffinization, genomic DNA from fresh and deparaffinized tissues was extracted using a High Pure FFPET DNA Isolation Kit (Roche Diagnostics Deutschland GmbH, Mannheim, Germany), according to the manufacturer’s instructions**.**

### MCPyV DNA detection by conventional polymerase chain reaction (PCR)

PCR was performed with 500 ng of DNA by the TEMPase Hot Start DNA Polymerase 2 × Master Mix (Ampliqon, Odense, Denmark) and 0.5 μM of each primer in a total volume of 25 μl. For detection of the MCPyV, viral protein 1 (*VP1*), large T antigen 1 (*LT1*), and *LT3* genes were used, as described previously^6^. The size of PCR-products was 440 bp, 309, and 352 bp for *LT1*, *LT3*, and *VP1*, respectively. Then, the PCR products were purified using QIAquick PCR Purification Kit (Qiagen, Hilden, Germany), according to the manufacturer’s instructions. PCR products were sequenced using an ABI automated sequencer (Applied Biosystems, Foster City, CA, USA).

MEGA version 6.0 software (http://www.megasoftware.net) was applied to analyze raw sequencing data. In order to confirm the presence of PCR-amplified DNA, the beta globin gene with the size of 110 bp was amplified.

### Determination MCPyV DNA viral load

The MCPyV DNA viral load was evaluated by LightCycler® 96 Real-Time PCR System (Roche Diagnostics Deutschland GmbH, Mannheim, Germany) with the PCR program and primer sequences, which were previously described^7^. The MCPyV DNA viral load was evaluated by dividing the viral DNA copy number by half of the RNase P gene copy number; each diploid cell had two copies of the RNase P gene. Plasmids for MCPyV *LT*-Ag and human RNase P gene (standard for real-time PCR) were prepared on the basis of previous research^8^. To draw a standard curve, real-time PCR was performed on a series of tenfold dilutions of the purified plasmids of MCPyV *LT*-Ag and RNase P, ranging from 2 × 10^1^ to 2 × 10^6^ copies/μl. In order to eliminate the possibility of contamination and false positive results, adjacent normal non-PTC samples were tested.

### Determination of MCPyV RNA expression

Total RNA from the PEBS and FBS sections was extracted with the RNeasy Kits (QIAGEN, CA, USA), according to the manufacturer’s instructions. The *LT*-Ag transcripts were amplified by using qualitative real-time reverse transcription PCR (real-time RT-PCR) to indicate the presence of MCPyV by using primers and PCR program, which were previously explained^9^. RNA extracted from MCC patients was used as a positive control. To confirm the specificity of the amplification, a melting curve analysis was performed on the PCR products.

### Statistical analysis

Data analysis was performed using IBM SPSS version 22.0 software (SPSS. Inc., Chicago, IL, USA). The Shapiro–Wilk test was utilized to assess the data normality of continuous data. Pearson's chi-square and Mann–Whitney U tests were also applied to assess quantitative variables and continuous variables, respectively. Two-tailed *P-*value less than 0.05 was considered statistically significant.

## Results

### Patient’s demographic and clinical characteristics

In total, 1057 PTC patients were enrolled in this study. The mean age of PTC patients and tumor size were 45.0 ± 13.3 years and 1.9 ± 1.0 mm, respectively. Overall, 275 (26.0%) and 782 (74.0%) patients were male and female, respectively. 502 (47.5%) patients (192 male and 310 female) were smoker. The clinical features and baseline demographics of patients with PTC are shown in Table 1.

**Table 1:** Demographic features in Iranian Patients with papillary thyroid cancer according to MCPyV *LT*-Ag positivity.

	MCPyV *LT*-Ag positivity		Total	*P*-value
	Positive	Negative		
Patients (No)	215 (20.3%)	842 (79.7%)	1057 (100.0%)	–
Mean age ± SD	52.4 ± 11.9	43.1 ± 12.9	45.0 ± 13.3	0.011*
Sex (male/female)	88/127 (40.9/59.1%)	187/655 (22.2/77.8%)	275/782 (26.0/74.0%)	< 0.001*
Smoking (yes/no)	89/126 (41.4/58.6%)	413/429 (49.1/50.9%)	502/551 (47.5/52.5%)	0.134
Tumor size (mean ± SD, mm)	2.2 ± 1.4	1.8 ± 0.9	1.9 ± 1.0	0.351
Multifocality (unifocal/multifocal)	96/119 (44.7/55.3%)	572/270 (67.9/32.1%)	668/389 (63.2/36.8%)	< 0.001*
Lymphovascular invasion (yes/no)	123/92 (57.2/42.8%)	147/695 (17.5/82.5%)	270/787 (25.5/74.5%)	< 0.001*
Extrathyroidal extension (yes/no)	109/106 (50.7/49.3%)	97/745 (11.5/88.5%)	206/851 (19.5/80.5%)	< 0.001*
Lymph node involvement (yes/no)	130/85 (60.5/39.5%)	301/541 (35.7/64.3%)	431/626 (40.8/59.2%)	< 0.001*
Capsular invasion (yes/no)	119/96 (55.3/44.7%)	382/460 (45.4/54.6%)	501/556 (47.4/52.6%)	0.009*

*SD* standard deviation, *mm* millimeter.

*Statistically significant (< 0.05).

### Identification of MCPyV Genome by Conventional PCR

Among the 1057 enrolled PTC patients, 412 (39.0%) and 645 (61.0%) samples were FBS and PEBS, respectively. 1057 PTC and 1057 adjacent non-PTC normal cells were tested by three primer sets (*LT1*, *LT3*, and *VP1*) by conventional PCR. MCPyV DNA was identified in 215 samples with the *LT3* region, 78 samples with the *LT1* region, and 24 samples with the *VP1* region. Of the 215 positive samples for *LT3* region, 17 samples were positive for both *LT1* and *VP1* regions. Of the 412 FBS, MCPyV DNA was identified in 126 samples with the *LT3* region, 110 samples with the *LT1* region, and 25 samples with the *VP1* region. Of the 89 PEBS samples, MCPyV DNA was detected in 89 samples with the *LT3* region, 32 samples with the *LT1* region, and one sample with the *VP1* region. In order to confirm the results of conventional PCR, the purified PCR products (*LT3* gene) were chosen for sequencing analysis. This sequence showed 100% homology with another Iranian isolate, MCV-*LT3* (GenBank: MF927967.1), MCV-*LT3* (GenBank: MF927968.1), and MCV-*LT3* (GenBank: MF927969.1).

Of the 1057 adjacent non-PTC normal samples, MCPyV DNA was found in 15 (1.4%) FBS with the *LT3* primers. The beta globin gene was consistently amplified in all samples.

### Comparison of MCPyV DNA viral load in FBS and PEBS

In this study, the PTC samples positive by conventional PCR with *LT3* primers were selected for quantitative real-time PCR to evaluate the viral load. Of the 1057 samples, the MCPyV *LT*-Ag was identified in 215 (20.3%) of subjects. Of those 215 positive samples, 88 (40.9%) and 127 (59.1%) of patients were males and females, respectively. The MCPyV positivity was statistically different between mean age (*P* = 0.011), sex (*P* < 0.001), multifocality (*P* < 0.001), lymphovascular invasion (*P* < 0.001), extrathyroidal extension (*P* < 0.001), lymph node involvement (*P* < 0.001), and capsular invasion (*P* = 0.009).

In general, MCPyV *LT*-Ag DNA load was quantified in 126 (30.6%) out of 412 FBS samples, and 89 (13.8%) out of 645 PEBS samples. MCPyV *LT*-Ag positivity in FBS compared to PEBS was significantly different between mean age (*P* = 0.002), lymphovascular invasion (*P* < 0.001), extrathyroidal extension (*P* = 0.019), and MCPyV positivity (*P* < 0.001) (Table 2).

**Table 2:** Comparison of demographic features between fresh and paraffin-embedded papillary thyroid cancer biopsy samples.

	Fresh biopsy samples (n = 412)	Paraffin-embedded biopsy sample (n = 645)	*P*-value
Mean age ± SD	46.5 ± 13.3	43.9 ± 13.2	0.002*
Sex (male/female)	117/295 (28.4/71.6%)	158/487 (24.5/75.5%)	0.158
Smoking (yes/no)	113/299 (27.4/72.6%)	389/256 (60.3/39.7%)	0.472
Tumor size (mean ± SD, mm)	2.1 ± 1.2	1.9 ± 0.9	0.065
Multifocality (unifocal/multifocal)	247/165 (59.9/40.1%)	421/224 (65.3/34.7%)	0.080
Lymphovascular invasion (yes/no)	143/269 (34.7/65.3%)	127/518 (19.7/80.3%)	< 0.001*
Extrathyroidal extension (yes/no)	95/317 (23.1/76.9%)	111/534 (17.2/82.8%)	0.019*
Lymph node involvement (yes/no)	176/236 (42.7/57.3%)	255/390 (39.5/60.5%)	0.304
Capsular invasion (yes/no)	204/208 (45.5/54.5%)	297/348 (46.0/54.0%)	0.271
MCPyV (positive/negative)	126/286 (30.6/69.4%)	89/556 (13.8/86.2%)	< 0.001*
MCPyV DNA viral load (mean ± SD)	2.3 × 10^–1^ ± 0.5 × 10^–1^	0.7 × 10^–4^ ± 0.1 × 10^–4^	< 0.001*

*SD* standard deviation, *mm* millimeter*.*

*Statistically significant (< 0.05).

In MCPyV-positive samples, the mean MCPyV copy number was higher in the patients with FBS (2.3 × 10^–1^ ± 0.5 × 10^–1^ copies/cell) compared to PEBS (0.7 × 10^–4^ ± 0.1 × 10^–4^ copies/cell), which this difference was statistically significant (*P* < 0.001) (Table 2).

The mean MCPyV copy number in adjacent non-PTC normal samples was 0.3 × 10^–5^ ± 0.02 × 10^–5^ copies/cell that was very lower than the cancerous samples.

### Comparison of RNA expression of MCPyV *LT*-Ag in FBS and PEBS

To study the relationship between MCPyV positivity and PTC, determination of viral DNA load alone was not adequate. Accordingly, in MCPyV DNA-positive PTC patients, *LT*-Ag and *VP1* genes expression was assessed at the MCPyV *VP1* and *LT*-Ag RNA level by real-time RT-PCR. Among the 215 positive MCPyV DNA samples, 122 (96.8%) FBS and 42 (47.2%) PEBS were adequate for RNA extraction. In FBS, 92 (75.4%) out of 122 samples and in 42 PEBS, only 11 (26.2%) samples expressed *LT*-Ag RNA; while *VP1* gene transcript was not detected in any samples. The *LT*-Ag RNA expression was higher than in FBS compared to PEBS.

## Discussion

So far, MCPyV is the only human polyomavirus to be linked to the cause of human cancer, MCC. The presence of the MCPyV genome in MCC tumors in the world has been indicated by several independent reports. Most studies have found that the detection rate of MCPyV in MCC is about 70–80%^6,10^. Previous reports have suggested a possible role of MCPyV in several cancers such as non-MCC cancers, lung, head and neck, cervical, and central nervous system tumors^8,11–13^.

The findings of our study indicated that the rate of cigarette smoking in women was more than men. Evidences are available that cigarette smoking may be correlated with thyroid cancer risk. Cho et al. in a cohort study demonstrated current smoking was correlated with a decreased risk of incident PTC in men but not in women^14^. The findings of pooled analysis of five prospective reports suggest that smoking is related to a 30–40% decreased risk of PTC^15^. Cigarette smoking could potentially affect the risk of thyroid cancer by changing sex steroid hormone level, serum thyroid antibodies, and thyroid stimulating hormone. Because of self-administered questionnaires have been used in all studies, selection and recall bias may have contributed to the null correlation^16^.

To the best of our knowledge, this is the first study that evaluated the relationship between MCPyV infection and PTC patients according to pathologic features. To date, few studies have evaluated the association of polyomavirus with thyroid cancer. In those studies, three of them detected sequences of simian vacuolating virus 40 (SV40) and the other one, BK in post-operative thyroid^17–20^.

However, realization of Koch’s four postulates has demonstrated difficult for any of the carcinogenic viruses discovered to date, the oncogenicity of polyomaviruses in thyroid cancer is still controversial. Genome integration, instead of the mere viral genome sequences or proteins identification, has been proposed as a means of elucidating the link between viruses and cancer, which could potentially provide answers^21^. More convincing evidence is definitely needed. The current study is just the beginning of a long research journey to clarify whether these viruses are the cause of the development of thyroid cancer or just represent innocent bystanders. However, this study indicated the relationship between MCPyV positivity and PTC.

In this study, we compared the MCPyV positivity and DNA viral load between FBS and PEBS samples. The findings of this study indicated that MCPyV DNA in 30.6% of FBS was positive, while the frequency of MCPyV DNA in PEBS was 13.8%. Also, the mean MCPyV DNA viral load was higher in the patients with FBS compared to PEBS, indicating a statistically significant difference. It seems that the quality of the samples was the reason for the differences in the number of positive samples by PCR with *LT3* primers and the genome copy number per cell. As a result, the FBS samples were suitable for detection of viral infection in PTC patients.

However, FBS are advantageous in the ways where the process is much faster compared to the PEBS process. While PEBS are not adequate for molecular analysis, FBS is very suitable for this issue. This is because of the PEBS preparation that affects the molecular data. FBS are also preferred in analyses such as next generation sequencing, mass spectrometry, and quantitative real-time PCR. Therefore, this type of samples is the gold standard for DNA and RNA analysis^22,23^.

The inability to detect MCPyV *LT*-Ag DNA and RNA in most positive PEBS, and the low number of DNA copies per cell, may be due to many reasons. First, it is well-known that DNA and RNA degradation occurs in PEBS, resulting in poor quality of DNA and RNA yields for routine and real-time PCR analysis^11,24^. Second, the low copy number of viral RNA and DNA in PEBS may indicate that MCPyV in these samples exists as a passenger virus without certain pathological findings. Third, MCPyV might play a role in cancer pathogenesis through the “hit-and-run” mechanism^8,12^. Viral hit-and-run oncogenesis scenarios indicate that transient acquisition of the genomes of oncoviruses can induce a permanent change in the gene expression pattern of the host cell, leading to malignant transformation^11^. In this case, viral genomes or small viral fragments may be found in malignant tumors or in the precursor stages of the tumor. The *T*-Ag expression of is necessary for growth of MCC cell lines in vitro, and in vivo has been established MCPyV as a causative factor in the MCC oncogenesis. Ironically, both the transformational functions and the mechanisms of hit and run in non-transforming viruses that appear to be unrelated to human cancer have been explored^11,25,26^.

To define MCPyV as another infectious agent correlated with PTC, DNA positivity of MCPyV alone is not enough to determine its etiology^9^. Several reports have indicated that the MCPyV *LT*-Ag expression is required for the oncogenesis of MCPyV-positive MCC^27,28^. In this case, we evaluated the *LT*-Ag gene expression at the RNA level. In our study, 75.4% of FBS and 26.2% of PEBS samples expressed *LT*-Ag RNA; while *VP1* gene transcript was not detected in any samples. Polyomaviruses replication, like MCPyV, shows an ordered gene expression cascade in which the *LT* gene transcript is expressed first as an early gene transcription, followed by the *VP1* gene expression as late gene transcription^29^. However, the loss of the viral replication ability is a common feature of virus-related tumors^30^. In most MCCs infected with MCPyV in which viral replication is disrupted, only the truncated *LT* gene is constitutively expressed, but not the *VP1* gene that cannot support replication (DNA binding domain is lacking) and the viral genome is integrated^6,31^. According to these findings, it is rational that we only found the *LT* RNA expression in PTC samples, but not the RNA expression of the *VP1* gene that reflects virus replication. To prove it, Hashida et al*.* used immunohistochemistry to evaluate the MCPyV *LT*-Ag expression and localization. The localization of powerful immunoreactivity in the tumor cell nuclei revealed the MCPyV *LT*-Ag expression in the lung cancer cells and also the genome integration of MCPyV into the host genome is considered as a key element in carcinogenesis^9^. The lack of performing immunohistochemistry especially for small *T*-Ag, truncated *LT*-Ag expression, and viral integration were the limitations of this study.

In this study, the MCPyV *LT*-Ag DNA loads were higher in cancerous cells than in non-cancerous samples. Several studies in different types of cancer showed the frequency of MCPyV DNA was significantly higher in the tumor cells than non-cancerous tissues^11,32^. This finding implies a transitory role for MCPyV in cell transformation, since its genome can be silenced or lost during cancer progression, which refers to the hit-and-run mechanism^33^.

In conclusion, the present study, for the first time, not only identified MCPyV DNA, but also the *LT* RNA transcripts expressions in PTC. Also, we indicated the viral DNA load in FBS was higher than in PEBS. However, more epidemiological and virological studies are required to determine the relationship between the pathogenicity of MCPyV with PTC.

## References

[1] Limaiem, F., Rehman, A. & Mazzoni, T. Papillary thyroid carcinoma. (2019). 30725628

[2] Mostafaei S (2020). Viral infections and risk of thyroid Cancer: a systematic review and empirical Bayesian meta-analysis. Pathol. Res. Pract..

[3] Dialameh PA (2021). Detection of human papillomavirus in papillary thyroid carcinoma and its association with tumor staging and pathologic features. Iran. J. Med. Sci..

[4] MacDonald M, You J (2017). Merkel cell polyomavirus: a new DNA virus associated with human cancer. Infect. Agents Assoc. Cancers Epidemiol. Mol. Biol..

[5] Liu W, You J (2020). Molecular mechanisms of merkel cell polyomavirus transformation and replication. Ann. Rev. Virol..

[6] Feng H, Shuda M, Chang Y, Moore PS (2008). Clonal integration of a polyomavirus in human Merkel cell carcinoma. Science.

[7] Becker JC (2008). MC polyomavirus is frequently present in Merkel cell carcinoma of European patients. J. Invest. Dermatol..

[8] Sadeghi F (2015). Detection of Merkel cell polyomavirus large T-antigen sequences in human central nervous system tumors. J. Med. Virol..

[9] Hashida Y (2013). Detection of Merkel cell polyomavirus with a tumour-specific signature in non-small cell lung cancer. Br. J. Cancer.

[10] DeCaprio JA (2017). Merkel cell polyomavirus and Merkel cell carcinoma. Philos. Trans. R. Soc. B Biol. Sci..

[11] Mohebbi E (2018). Low viral load of Merkel cell polyomavirus in Iranian patients with head and neck squamous cell carcinoma: Is it clinically important?. J. Med. Virol..

[12] Behdarvand A (2017). Evaluation of Merkel cell polyomavirus in non-small cell lung cancer and adjacent normal cells. Microb. Pathog..

[13] Csoboz B (2020). Merkel cell polyomavirus and non-Merkel cell carcinomas: Guilty or circumstantial evidence?. APMIS.

[14] Cho A (2018). Cigarette smoking and thyroid cancer risk: A cohort study. Br. J. Cancer.

[15] Kitahara CM (2012). Cigarette smoking, alcohol intake, and thyroid cancer risk: A pooled analysis of five prospective studies in the United States. Cancer Causes Control.

[16] Soldin OP (2009). Thyroid hormone levels associated with active and passive cigarette smoking. Thyroid.

[17] Pacini F (1998). Simian virus 40-like DNA sequences in human papillary thyroid carcinomas. Oncogene.

[18] Vivaldi A (2003). Simian virus 40-like sequences from early and late regions in human thyroid tumors of different histotypes. J. Clin. Endocrinol. Metab..

[19] Ozdarendeli A (2004). SV40 in human thyroid nodules. J. Clin. Virol..

[20] Stamatiou D (2015). Investigation of BK virus, Epstein-Barr virus and human papillomavirus sequences in postoperative thyroid gland specimens. Int. J. Biol. Markers.

[21] Sarid R, Gao S-J (2011). Viruses and human cancer: from detection to causality. Cancer Lett..

[22] Gao XH (2020). Comparison of fresh frozen tissue with formalin-fixed paraffin-embedded tissue for mutation analysis using a multi-gene panel in patients with colorectal cancer. Front. Oncol..

[23] Lüder Ripoli F (2016). A comparison of fresh frozen vs formalin-fixed, paraffin-embedded specimens of canine mammary tumors via branched-DNA assay. Int. J. Mol. Sci..

[24] Hattori T (2013). The prevalence of Merkel cell polyomavirus in Japanese patients with Merkel cell carcinoma. J. Dermatol. Sci..

[25] Niller HH, Wolf H, Minarovits J (2011). Viral hit and run-oncogenesis: genetic and epigenetic scenarios. Cancer Lett..

[26] Houben R (2012). Merkel cell carcinoma and Merkel cell polyomavirus: evidence for hit-and-run oncogenesis. J. Investig. Dermatol..

[27] Houben R (2012). An intact retinoblastoma protein-binding site in Merkel cell polyomavirus large T antigen is required for promoting growth of Merkel cell carcinoma cells. Int. J. Cancer.

[28] Sihto H (2011). Merkel cell polyomavirus infection, large T antigen, retinoblastoma protein and outcome in Merkel cell carcinoma. Clin. Cancer Res..

[29] Feng H (2011). Cellular and viral factors regulating Merkel cell polyomavirus replication. PLoS ONE.

[30] Zur Hausen H (2008). A specific signature of Merkel cell polyomavirus persistence in human cancer cells. Proc. Natl. Acad. Sci..

[31] Shuda M (2008). T antigen mutations are a human tumor-specific signature for Merkel cell polyomavirus. Proc. Natl. Acad. Sci..

[32] Yahyapour Y, Sadeghi F, Alizadeh A, Rajabnia R, Siadati S (2016). Detection of merkel cell polyomavirus and human papillomavirus in esophageal squamous cell carcinomas and non-cancerous esophageal samples in northern Iran. Pathol. Oncol. Res..

[33] Jung WT, Li MS, Goel A, Boland CR (2008). JC virus T-antigen expression in sporadic adenomatous polyps of the colon. Cancer.

